# Healthcare professionals’ perceived barriers in providing palliative care in primary care and nursing homes: a survey study

**DOI:** 10.1177/26323524231216994

**Published:** 2023-12-25

**Authors:** Katrin Kochems, Everlien de Graaf, Ginette M. Hesselmann, Marieke J. E. Ausems, Saskia C. C. M. Teunissen

**Affiliations:** Center of Expertise in Palliative Care, Julius Center for Health Sciences and Primary Care, University Medical Center Utrecht, Heidelberglaan 100, P.O. Box 85500, Utrecht 3508 GA, The Netherlands; Center of Expertise in Palliative Care, Julius Center for Health Sciences and Primary Care, University Medical Center Utrecht, Utrecht, The Netherlands; Cancer Center, University Medical Center Utrecht, Utrecht, The Netherlands; Academic Hospice Demeter, De Bilt, The Netherlands; Center of Expertise in Palliative Care, Julius Center for Health Sciences and Primary Care, University Medical Center Utrecht, Utrecht, The Netherlands

**Keywords:** multidimensional care, multiprofessional care, nursing home, palliative care, primary care, survey

## Abstract

**Background:**

Palliative care in primary care and nursing home settings is becoming increasingly important. A multidimensional palliative care approach, provided by a multiprofessional team, is essential to meeting patients’ and relatives’ values, wishes, and needs. Factors that hamper the provision of palliative care in this context have not yet been fully explored.

**Objectives:**

To identify the barriers to providing palliative care for patients at home or in nursing homes as perceived by healthcare professionals.

**Design:**

Cross-sectional survey study.

**Methods:**

A convenience sample of nurses, doctors, chaplains, and rehabilitation therapists working in primary care and at nursing homes in the Netherlands is used. The primary outcome is barriers, defined as statements with ⩾20% negative response. The survey contained 56 statements on palliative reasoning, communication, and multiprofessional collaboration. Data were analyzed using descriptive statistics.

**Results:**

In total, 249 healthcare professionals completed the survey (66% completion rate). The main barriers identified in the provision of palliative care were the use of measurement tools (43%), consultation of an expert (31%), estimation of life expectancy (29%), and documentation in the electronic health record (21% and 37%). In primary care, mainly organizational barriers were identified, whereas in nursing homes, most barriers were related to care content. Chaplains and rehabilitation therapists perceived the most barriers.

**Conclusion:**

In primary care and nursing homes, there are barriers to the provision of palliative care. The provision of palliative care depends on the identification of patients with palliative care needs and is influenced by individual healthcare professionals, possibilities for consultation, and the electronic health record. An unambiguous and systematic approach within the multiprofessional team is needed, which should be patient-driven and tailored to the setting.

## Introduction

The need for palliative care is increasing in primary care and nursing homes as 41% of people indicative of palliative care needs die at home and 27% in nursing homes.^1^ Palliative care aims to optimize the quality of life of patients facing life-threatening illness and their families.^2,3^ To meet the values, wishes, and needs of patients and their relatives, a multidimensional approach provided by a multiprofessional team of healthcare professionals (HCPs) is needed.

Palliative care addresses the patient’s holistic well-being, which encompasses physical, psychological, social, and spiritual dimensions.^4–7^ For optimal palliative care, multidimensional symptoms and concerns should be managed in a cyclic process of signaling, analysis and treatment, monitoring symptom intensity, evaluating the treatment effect, and adjusting individual care plans if needed.^8,9^ The assessment and management of symptoms and concerns as a multidimensional experience underlines the significance of a multiprofessional approach in addressing multidimensional care needs.^6,8,10^ Thus an interprofessional collaboration between members of the multiprofessional team, communication, and coordination is essential for the continuity of treatment and an optimal therapeutic effect^10–12^ as well as informed and shared decision-making.^6,13^ Other fundamental elements comprise a systematic and proactive approach,^8,10,12^ and documentation in the health record.^10,14^

In practice, the palliative care provided is often inadequate for patients cared for at home^14,15^ and in nursing homes.^7,14,16–19^ Symptoms prevalence is high in all four dimensions,^5,20^ symptoms are often assessed and managed inadequately,^7^ or they remain untreated.^15,17^ Untreated symptoms concerned mostly non-pain and psychological symptoms and social and spiritual concerns, implying that multidimensional symptom management is not performed optimally.^5,7,21^ In addition, many patients do not receive palliative care although they are eligible for it,^18^ resulting in poor quality of dying.^15,22,23^

The Netherlands is a country with a generally high-level development of palliative care,^24^ with palliative care being part of the governments’ health policy since 2007. Despite this, patients report suboptimal palliative care and reduced quality of life,^16^ insufficient and limited communication, and multiprofessional collaboration between HCPs.^25^ Palliative care is lacking structural incorporation into education, and the delivery model involves both generalists and specialized professionals.^3,26^ To improve availability and access to high-quality palliative care, the Netherlands Quality Framework for Palliative Care was developed.^3^ This framework promotes interprofessional collaboration in the form of multiprofessional meetings and structured clinical documentation. However, the application of this collaboration varies among the settings. The frequency of multiprofessional meetings within nursing homes varies, but they are held at least once every 6 months. In primary care, there is collaboration between general practitioners (GPs) and district nurses (DNs), which is called PaTz (an acronym for ‘Palliatieve Thuiszorg’; palliative care at home),^27^ but it is not implemented nationally. Transparent electronic medical record systems are not kept uniformly, resulting in challenges in communication and the duplication of documentation.

Given the increasing need for care for patients in the palliative phase, cared for at home and in nursing homes, it is important to identify barriers to providing palliative care. Based on the Netherlands Quality Framework for Palliative Care, this study focuses on the essentials of palliative care as well as areas known to need improvement to provide high-quality palliative care: the structure and process of palliative care (palliative reasoning), communication with patients and relatives, and multiprofessional collaboration.^3^ It is common knowledge that barriers in these aspects exist,^25^ but specific insights into knowledge, skills, and attitudes of all involved HCPs working in primary care and nursing homes are lacking. A better understanding of HCP-perceived barriers and their origin can guide interventions to improve palliative care provision. Thus, the aim of this study is to identify barriers to providing palliative care in primary care and nursing homes, where most palliative care patients reside.

## Methods

### Study design

A cross-sectional survey study was conducted between June and October 2019 with multidisciplinary HCPs working in primary care or nursing homes. The ‘Strengthening the Reporting of Observational Studies in Epidemiology (STROBE) statement: guidelines for reporting observational studies’ was used.^28^

### Setting and participants

A convenience sample of collaboration between GPs and DNs, called PaTz,^27^ and nursing homes in the center of the Netherlands were recruited. Settings were eligible for inclusion if the chair or the management expressed a motivation for participation and if there was a willingness to facilitate the study procedures. All HCPs from each PaTz group and nursing home included were invited. Settings were contacted for participation by an e-mail, which included an informative letter about the study. A contact person or the investigator distributed the survey invitation, including the survey link, to all HCPs *via* e-mail. Additionally, HCPs from PaTz groups were asked to distribute the survey within their network. HCPs were eligible if they were professional caregivers providing care to patients in the last year of life.

### Data collection

Data were collected using the open survey software SurveyMonkey.^29^ A reminder was sent 3–6 weeks after the initial invitation, depending on the contact person. The survey software automatically collected all responses. Participants could access the online survey *via* a survey link provided to them in the invitation e-mail at their convenience.

### Survey instrument

A survey instrument was developed to identify barriers to providing palliative care in primary care and nursing homes. Survey items were based on three domains: (1) palliative reasoning, (2) communication with patients and relatives, and (3) multiprofessional collaboration. Statements on the domain of palliative reasoning were based on a palliative-care-adapted clinical reasoning approach to optimize the systematic attention for all dimensions following an iterative process: (1) assessment of the individual situation of the patient, (2) summary of the problem and formulation of a proactive care plan, (3) evaluation, and (4) modification of the care plan as needed and constant evaluation.^9,30^ Statements on the domains of communication with patients and relatives and multiprofessional collaboration were based on the Netherlands Quality Framework for Palliative Care^3^ and supported by relevant literature.^25,31^ Multiprofessional collaboration was further divided into sub-domains: (a) palliative reasoning, (b) acknowledgment within the multiprofessional team, (c) multiprofessional consultations, and (d) documentation in the electronic health record.

Domains included statements (domain 1: *n* = 21; domain 2: *n* = 6; domain 3: *n* = 26) that participants could rate using a five-point Likert-type scale, ranging from either ‘always’ to ‘never’, ‘strongly agree’ to ‘strongly disagree’, or ‘excellent’ to ‘inadequate’. In addition, three questions were answered with ‘yes’ or ‘no’ (domain 3). If appropriate, questions included answer possibilities such as ‘not applicable’, ‘unknown’, or ‘otherwise, namely’. Answers on a five-point Likert-type scale were summarized into three categories: positive, negative, and non-informative. Bottlenecks were defined as all statements where ⩾20% of participants answered negatively, according to the categorized statements.

Participants’ characteristics included gender, age, profession, years of working experience in the profession, working hours per week, and additional education or training in palliative care. In addition, the surveys for primary care included questions on PaTz group membership. At the beginning of the survey, participants were asked if they provided palliative care; if they answered no, the survey ended for them.

Survey items were based on the expertise of the multidisciplinary research team. Face and content validity were tested after consultation with experts working in the field of palliative care. After three stages of development, in which the survey was pilot-tested among the target population (*n* = 23) and experts (*n* = 5), suggested changes, such as wording to improve comprehensibility, were discussed with the research team and changed after a consensus was reached. The final version was one survey with six modules modified per target population and setting.

### Data analysis

Data were analyzed using descriptive statistics. Bottlenecks that were identified were stratified by setting and profession, and the characteristics of all participants were summarized. Data were exported from SurveyMonkey into Microsoft Office Excel version 2016^32^ and analyzed. Participants who responded with ‘no’ to the question about the provision of palliative care were excluded from data analysis. Duplicate respondents were checked based on IP addresses and removed.

## Results

### Characteristics of participants

**Figure 1.**
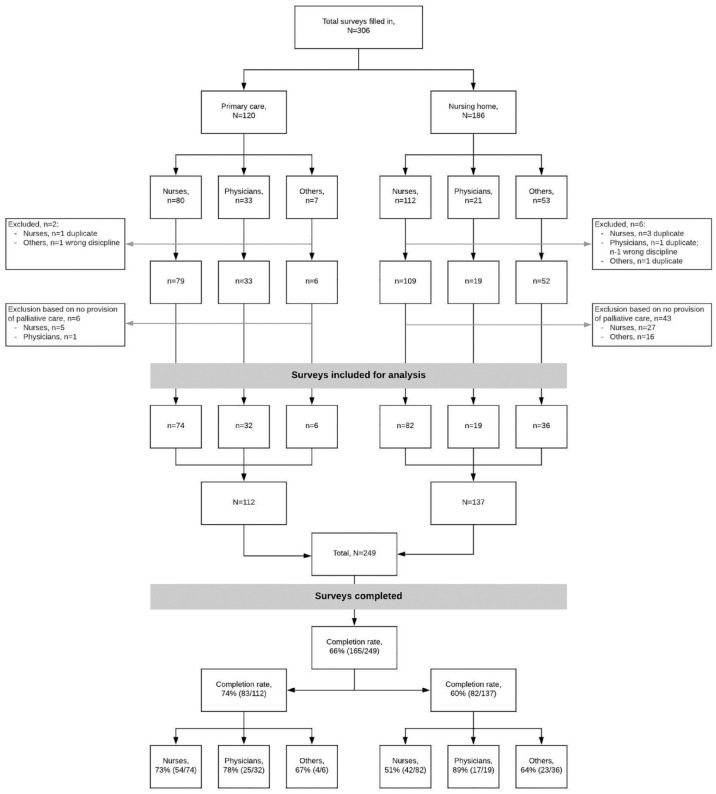
Flowchart of participation.

In total, 22 PaTz groups and 13 nursing homes were invited, of which 5 (39%) and 11 (50%) agreed to participate, respectively. Reasons for nonparticipation were time-related. In total, 306 surveys were completed. After the exclusion of 57 participants: wrong discipline (*n* = 2),duplicates (*n* = 6), and not providing palliative care (*n* = 49), 249 participants were included for analysis: 112 from primary care and 137 from a nursing home (Table 1). Of the 249 participants, 165 completed the survey, resulting in a completion rate of 66% (74% primary care, 60% nursing home; Figure 1). The majority of participants were female (89%), with a sample mean age of 45 years (±13). The total sample had a median work experience in their profession of 11 years and worked a weekly median of 28 h. In the primary care sample, 66% were nurses (62% nurses and 4% nurse assistants), 29% physicians, 4% chaplains, and 2% rehabilitation therapists. In the nursing home sample, 60% were nurses (36% nurse assistants and 23% nurses), 23% rehabilitation therapists, 14% physicians, and 3% chaplains. Training in palliative care during initial education was received by 21% of primary care respondents and 29% of nursing home respondents; nurses and physicians in the nursing home did not follow any additional specialized education in palliative care compared to nurses and physicians from primary care (13% primary care, 0% nursing home). Furthermore, respondents in primary care followed occasional courses on palliative care more often (68% primary care, 40% nursing home).

**Table 1.** Participant characteristics.

Characteristics	Primary care				Nursing home				Total
	Nurses	Physicians	Others	Total	Nurses	Physicians	Others	Total	
	*N* = 74	*N* = 32	*N* = 6	*N* = 112	*N* = 82	*N* = 19	*N* = 36	*N* = 137	*N* = 249
Gender, female, *n* (%)	73 (99)	21 (66)	5 (71)	98 (88)	78 (95)	14 (74)	32 (89)	127 (93)	222 (89)
Age, mean (SD)	44 (13)	49 (7)	56 (13)	46 (12)	42 (13)	44 (12)	44 (15)	43 (13)	45 (13)
Profession, *n* (%)									
Nurse assistant	5 (7)			5 (4)	50 (61)			50 (36)	55 (22)
Registered nurse	18 (24)			18 (16)	28 (34)			28 (20)	46 (18)
Specialized nurse in palliative care/oncology	11 (15)			11 (10)	4 (5)			4 (3)	15 (6)
District nurse	40 (54)			40 (36)	0 (0)			0 (0)	40 (16)
Basic physician		0 (0)		0 (0)		2 (11)		2 (1)	2 (1)
Elderly-care physician		1 (3)		1 (1)		15 (79)		15 (11)	16 (6)
General practitioner		28 (88)		28 (25)		2 (11)		2 (1)	30 (12)
Physician specialized in palliative care		3 (9)		3 (3)		0 (0)		0 (0)	3 (1)
Chaplain			4 (67)	4 (4)			4 (11)	4 (3)	8 (3)
Occupational therapist				0 (0)			10 (28)	10 (7)	10 (4)
Speech therapist				0 (0)			3 (8)	3 (2)	3 (1)
Physiotherapist			2 (33)	2 (2)			10 (28)	10 (7)	12 (5)
Dietitian				0 (0)			1 (3)	1 (0)	1 (0)
Psychologist				0 (0)			8 (22)	8 (6)	8 (3)
Years working in profession, median (IQR)	6 (14)	19 (12)^a^	14 (25)	10 (16)	12 (18)^b^	13 (13)	11 (13)	13 (17)	11 (15)
Weekly working hours, median (IQR)	28 (8)^c^	36 (10)	16 (11)^d^	28 (8)	26 (12)^b^	32 (7)^e^	28 (9)^f^	28 (9)	28 (8)
Palliative care education, yes, *n* (%)									
During initial education	15 (20)	6 (19)	2 (33)	23 (21)	24 (29)	10 (53)	6 (31)	40 (29)	63 (25)
Additional specialized education^g^	8 (11)	6 (19)	NA	NA	0 (0)	0 (0)	NA	NA	NA
Occasional courses^h^	51 (69)	22 (69)	3 (50)	76 (68)	35 (43)	9 (47)	11 (31)	55 (40)	131 (53)
Member of PaTz group, *n* (%)	47 (64)	31 (97)	3 (50)	81 (72)	NA	NA	NA	NA	NA

a *n* = 2 missing (based on *n* = 30 responses).

b *n* = 3 missing (based on *n* = 79 responses).

c *n* = 4 missing (based on *n* = 70 responses).

d *n* = 1 missing (based on *n* = 5 responses).

e *n* = 1 missing (based on *n* = 18 responses).

f *n* = 5 missing (based on *n* = 31 responses).

g For nurses: post-higher vocational education; for physicians: specialization course palliative care.

h Additional courses within the own organization; courses given by external organizations; congress/symposia.

IQR, interquartile range; NA, not applicable; PaTz, Palliatieve Thuiszorg; SD, standard deviation.

### Barriers to providing palliative care

**Figure 2.**
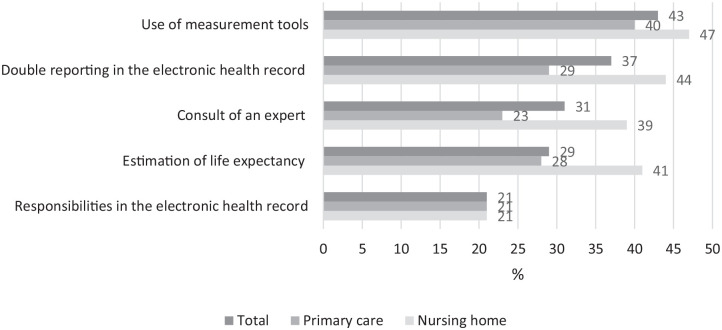
Barriers to providing palliative care.

Barriers were identified in the domains of *palliative reasoning* and *documentation in the electronic health record* (Figure 2; Table 2). Estimating the life expectancy (29%), considering consultation of an expert and/or performance of additional diagnostics (31%), and use of measurement tools (43%) were identified as barriers. Within the documentation of the electronic health record, HCPs stated that double reporting is not avoided (37%) and that responsibilities are not clear (21%).

**Table 2.** Identified barriers in total and stratified by setting and profession.

Barriers (statements/questions with ⩾20% negative response)	Total		Primary care								Nursing home							
			Total		*N*		*P*		C/RT		Total		*N*		*P*		C/RT	
	%	*n*/*N*	%	*n*/*N*	%	*n*/*N*	%	*n*/*N*	%	*n*/*N*	%	*n*/*N*	%	*n*/*N*	%	*n*/*N*	%	*n*/*N*
Palliative reasoning (sometimes/never)																		
Step 1: Assess the individual situation																		
Medical history																	28	18/29
Physical dimension																	24	9/29
Psychological dimension																		
Social dimension																	21	6/29
Spiritual dimension															29	5/17	21	6/29
Estimate life expectancy	29	63/214	28	20/108	32	23/72			50	3/6	41	43/106	42	25/60			55	16/29
Medication inventory											25	27/106					59	17/29
Symptom analysis									50	3/6							34	10/29
Meaning of the symptom for the patient											22	23/106			24	4/17	38	11/29
Priorities and wishes of the patient																		
Step 2: Summarize the problem and formulate a proactive care plan																		
Name the problems and formulate a working hypothesis									33	1/6							34	5/29
Formulate an individual care plan	22	47/214							50	3/6	26	28/106	28	17/60			38	11/29
Consider consultation with an expert and/or performing additional diagnostics	31	66/214	23	25/108	22	16/72	20	6/30	50	3/6	39	41/106	30	18/60	47	8/17	52	15/29
Formulate aims of treatment							20	6/30	50	3/6	23	24/106	25	15/60			28	8/29
Formulate a proactive care plan											32	34/106	30	18/60			52	15/29
Step 3: Evaluate																		
Plan how the effect of the care plan is measured, by whom, and when	27	57/214					43	13/30			31	33/106	20	12/60	29	5/17	55	16/29
Measure the effect by symptom severity, experience, patient functioning, and well-being			22	11/108					33	2/6								
Step 4: Adapt the care plan as needed and constant evaluation																		
Adapt the care plan as needed and constant evaluation			27	10/108					33	2/6							21	6/29
At each step																		
Coordinate with the patient and relatives																		
Use of measurement tools	43	93/214	40	43/108	29	21/72	63	19/30	50	3/6	47	50/106	30	18/60	88	15/17	59	17/29
Counseling of relatives																		
Communication with patients and relatives (sometimes/never)																		
Involving the patient/relatives when drawing up the care plan for admission/start of care																	25	6/24
Including wishes/priorities of the patient/relatives on all four dimensions when creating the care plan									25	1/4								
Informing patients/relatives about changes in care if the care plan is modified																	25	6/24
Providing the opportunity to discuss patient/relatives’ needs and priorities on all four dimensions at any time																		
Engaging in conversation with the patient/relatives if I notice a change in him or her																		
Actively involving the patient/relatives in the decision-making process surrounding treatment/care																		
Multiprofessional collaboration																		
Palliative reasoning (sometimes/never or mediocre/inadequate)																		
Understanding between HCPs from different disciplines when a patient’s problems/symptoms are charted																		
Communication between HCPs in identifying a patient’s problems/symptoms									25	1/4							26	6/23
Involvement in decision-making regarding the patient’s care plan	29	36/125					NA	NA	50	2/4	39	26/66	26	11/43	NA	NA	66	15/23
Involvement in the implementation of patient care	22	31/142					NA	NA	75	3/4	27	22/83			30	5/17	61	14/23
Acknowledgment within the multiprofessional team (disagree/strongly disagree)																		
Heard by fellow team members																		
Respected by fellow team members																		
Safe to express myself																		
Strong in communication with fellow team members about knowledge																		
Safe to discuss patient care issues																		
Understood by fellow team members																		
Equal in collaboration									25	1/4								
Appreciated by my fellow team members in my work																		
Multiprofessional consultations (no)																		
Multiprofessional consultations are conducted			20	18/88			38	10/26	25	1/4								
If conducted, participation in consultations											24	20/84	39	17/44				
Planned consultations (of all consultations conducted)	35	51/146	62	40/65														
Documentation in the electronic health record (disagree/strongly disagree)																		
The structure is good																	23	5/23
The content is appropriate																		
The method is consistent with daily care																		
There are options to add items					21	10/48							21	9/44			23	5/23
Supports care process																	23	5/23
Supports mutual communication							38	10/26	33	1/3							27	6/23
Allows space to note specific points of interest																		
Double reporting is avoided	37	59/161	29	22/77			46	12/26	33	1/3	44	37/84	36	16/44	47	8/17	59	13/23
Makes information sharing between HCP easier	26	42/161	35	27/77	26	12/48	54	14/26	33	1/3							23	5/23
A patient’s care process is easy to follow																		
Efficient									33	1/3	27	23/84	25	11/44			41	9/23
Responsibilities are clear	21	34/161	21	16/77	21	10/48	23	6/26			21	18/84			24	4/17	32	7/23
Supports multiprofessional collaboration	27	43/161	38	29/77	31	15/48	50	13/26	33	1/3							32	7/23
Supports problem analysis and decision-making	20	33/161	23	18/77			42	11/26									23	5/23

C/RT, chaplains/rehabilitation therapists; HCP, healthcare professionals; N, nurses; NA, not applicable; P, physicians.

### Barriers stratified by setting

#### Primary care

**Figure 3.**
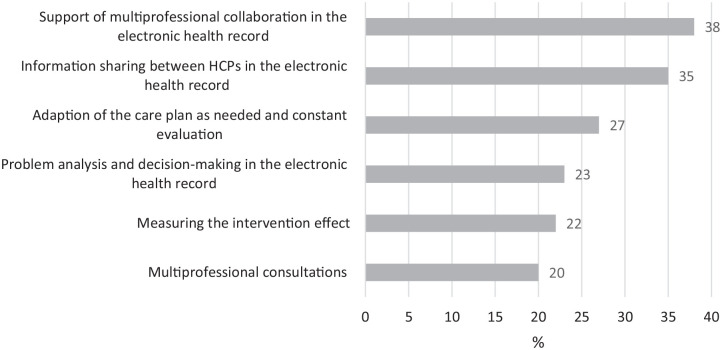
Barriers to providing palliative care in primary care.

Barriers identified specifically in primary care were found in *palliative reasoning* and *multiprofessional collaboration* (Figure 3). The effect of interventions on symptom severity, experience, patient functioning, and well-being is sometimes or never assessed 22% of the time. Of the participants, 27% sometimes or never adapt the care plan as needed and evaluate constantly. Furthermore, multiprofessional consultations are not conducted in each setting (20%), and these are mostly not planned (62%). Barriers concerning the documentation in the electronic health record concerned that information sharing between HCPs was not made easier (35%), and did not support multiprofessional collaboration (38%) nor problem analysis and decision-making (23%).

#### Nursing home

**Figure 4.**
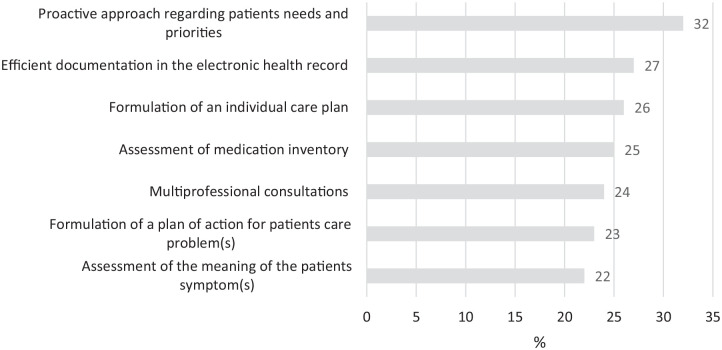
Barriers to providing palliative care in nursing homes.

In nursing homes, most barriers were identified within the domain of *palliative reasoning* (Figure 4): assessment of medication inventory (25%) and the meaning of the symptom for the patient (22%), formulation of an individual care plan (26%) and a plan of action for patient’s care problem(s) (23%), and a proactive approach regarding patient’s needs and priorities (32%). If multiprofessional consultations are conducted, 24% of all HCPs did not participate. Lastly, 27% of HCPs stated that documentation in the electronic health record is not efficient.

### Stratified by profession

In both settings, chaplains and rehabilitation therapists perceived the most barriers, followed by physicians and nurses in primary care and nurses and physicians in nursing homes (Table 2). Although most physicians followed the steps of palliative reasoning in practice, this is less the case for nurses in nursing homes and chaplains and rehabilitation therapists in both settings; chaplains and rehabilitation therapists in nursing homes identified almost all steps of palliative reasoning as a barrier. It is also striking that physicians in particular do not use measurement tools (63% primary care, 88% nursing home). Furthermore, the inclusion of patients’ wishes and needs was problematic for chaplains and rehabilitation therapists in both settings (25%). Chaplains and rehabilitation therapists in nursing homes identified barriers to involving the patient/relative when drawing up the care plan for admission/start of care and informing patients/relatives about changes in care (25%). Concerning *the multiprofessional collaboration*, chaplains and rehabilitation therapists from both settings stated that the communication between HCPs in identifying a patient’s problems/symptoms is inadequate (25% primary care, 26% nursing home), that they are not always involved in decision-making regarding the patient’s care plan (50% primary care, 66% nursing home) and implementation of patient care (75% primary care, 61% nursing home). Furthermore, 25% of chaplains and rehabilitation therapists in primary care perceived barriers in feeling equal in collaboration. Over one-third of nurses in nursing homes perceived barriers to participating in multiprofessional consultations (39%). Documentation in the electronic health records forms a barrier for chaplains and rehabilitation therapists in nursing homes, followed by physicians in primary care and chaplains and rehabilitation therapists in primary care.

## Discussion

This study showed that, in both primary care and nursing homes, barriers exist to the provision of palliative care. Not all HCPs implement steps necessary for optimal palliative care, such as estimating life expectancy, the use of measurement tools, and consultation with an expert. Furthermore, multiprofessional collaboration about multiprofessional consultations and documentation in the electronic health record does not support the provision of palliative care. In primary care, barriers were mainly indicated on an organizational level, whereas, in nursing homes, barriers were mostly related to care content. Chaplains and rehabilitation therapists perceived the most barriers.

## Care content-related barriers

In both settings, participants do not pay systematic attention to symptoms indicated by following all the steps of palliative reasoning, which is needed for a comprehensive palliative care approach.^9,30^ Although more barriers were identified within the nursing home setting, the estimation of life expectancy was identified as a barrier in both settings. The identification of patients with possible palliative care needs depends on the estimation of prognosis, and if not optimally performed, this increases the risk of not identifying patients with palliative care needs, resulting in undertreatment or overtreatment and a lack of multidimensional support.^3,33^ This barrier may also be reflected in the number of nursing home respondents excluded from this study because they stated that they do not provide palliative care (*n* = 43). Furthermore, prognostic uncertainty hampers HCPs from initiating end-of-life conversations with patients and relatives.^34^ Patients need caring and effective communication to make well-informed decisions for (future) care.^35,36^ In this study, HCPs did not perceive barriers in communication with patients and relatives, although poor communication or a lack of communication between HCPs and patients or relatives is identified as a barrier to providing and consequently using palliative care in other studies.^37–39^ Patients often perceive communication with HCPs as ineffective due to a lack of communication skills, use of language unfamiliar to the patient, HCPs that do not listen, and not being taken seriously.^37,40–42^ HCPs from this study seem to not be aware of or experience these perceptions. The discrepancy in perceptions exposes the necessity of communication and requires attention for further research.

It was also striking that 43% of all participants did not use measurement tools. To treat symptoms, they have to be identified. A systematic approach to assessment and monitoring identifies more symptoms.^43,44^ For both patients and HCPs, it is not always natural to express palliative care needs in words and to discuss them.^45^ Patient-reported outcome measures (PROMS) can support HCPs in identifying symptoms and needs of patients and monitoring their severity. Furthermore, these instruments can facilitate a conversation about the meaningfulness of symptoms experienced by the patient.^46^ To provide palliative care, HCPs working in primary care and nursing homes need support in using instruments to measure symptoms.^47^

These identified HCP-related barriers, estimation of life expectancy, and the use of measurement tools may indicate a lack of knowledge and insufficient education, since other studies showed that poor knowledge, inadequate education, or experience in palliative care hampered HCPs in implementing a palliative approach.^37,38,48^

These care content-related barriers were more evident in the nursing home setting, which could be due to the organization of care. In nursing homes, medical, paramedic, psychological, and social care is integrated with nursing,^49^ leading to a wide variety of educational levels within the team. Elderly-care physicians, who specialize in elderly-care medicine,^50^ are responsible for medical care and care plans,^49^ while it is predominantly nurse assistants who provide daily care. In addition to the elderly-care physician, nurse assistants, and registered nurses/nurse specialists, the nursing home team consists of various disciplines such as physiotherapists, psychologists, social workers, and chaplains.^49^ Since nurse assistants and nurses provide daily care, they have an important role in identifying symptoms, wishes, and needs of patients and relatives.^51^ However, several studies showed that skills and knowledge of palliative care are minimal^47,52,53^ and that education of nursing staff needs to be improved,^52,54–56^ since this may negatively affect the quality of palliative care.^57–59^ Furthermore, elderly-care physicians stated that they lack competence in palliative care and that they need more education,^52^ and that national standardization of methodologies and palliative care tools and systems are lacking.^52,60^ These findings conflict with the complexity of nursing home care, which is rapidly increasing due to a decrease in the length of stay, an increase in care and support needs, and more severe problems.^60^ Most nursing home residents are in their final months or years of life,^61^ remain in the nursing home until death^49,62^ and could benefit from palliative care.

## Organizational barriers

Moreover, barriers were identified in consulting an expert, multiprofessional consultations, and the electronic health record – all aspects that may indicate inefficient interprofessional collaboration between HCPs. Within the interprofessional collaboration, all HCPs involved combine their competencies to care for patients, which can benefit organizations, professionals, and patients when collaboration takes place highly effectively.^63,64^ The multidimensional approach requires the knowledge and involvement of several professionals working together in close collaboration with the patient and relatives.^3^ Within this collaboration, HCPs involved evaluate care at set times, HCPs specialized in palliative care will be consulted when HCPs involved cannot meet the values, wishes, and needs of the patient and relatives, and HCPs work with a shared and continuously updated electronic health record to sustain collaboration.^3^ Collaboration and communication between HCPs were also identified as a barrier in other studies, which was explained by a poor exchange of information, a lack of effective team communication and opportunities to communicate, and a lack of effective ways to give and receive patient care information.^37,64^

These barriers were more prevalent in the primary care setting. Differences in findings between both settings can be explained by the organization of settings. In primary care, multidisciplinary collaboration is not inherently present. The GP and the DN play a leading role in the provision and coordination of palliative care, although collaboration remains difficult due to the lack of structural consultations, a collaborative care plan with a focus on continuity and evaluation, and insightful reporting of care.^65^ Communication and collaboration were experienced as increasingly difficult when more HCPs were involved and when the situation of the patient was complex and changing rapidly.^65^ To optimize and ameliorate collaboration in primary care, PaTz groups were developed, where GPs and DNs meet regularly to identify patients with palliative care needs and to discuss care for these patients.^66^ A pre- and post-survey study showed that the implementation of multiprofessional collaboration improved the provision of palliative care.^66,67^ Although GPs and DNs are the main providers of palliative care, if needed, they can refer to a chaplain, rehabilitation therapist, or social worker. However, in practice, referrals are rare and thus involvement is not yet standard.^68,69^ From the perspective of chaplains, noninvolvement in palliative care provision was mainly based on the lack of structural funding, knowledge of spiritual care by other HCPs, and how to find them.^70^ Multiprofession consultations were mentioned as a possible solution by GPs, DNs, and chaplains.^69,70^

Other interventions and innovative models of collaborative palliative care that involve different HCPs are needed to improve interprofessional collaboration between HCPs and therefore multidemensional care. Concrete examples of such interventions and innovative models are telehealth services, such as videoconferencing, telephonic communication, or remote symptom monitoring,^71^ interprofessional collaboration in teleconsultations,^72^ such as telephone-based collaborative care,^73^ and regionally organized collaborative palliative care.^74^ Several studies showed that this care improved patients’ quality of life, reduced emergency department visits and the risk of dying in the hospital, the access to HCPs, and decreased caregiver depression and burden.^73–76^ Interventions support HCPs to assess patient care needs,^71,77^ to optimize information flow^77^ and can improve interprofessional collaboration.^72^ HCPs experienced this collaborative model as positive, with improved knowledge exchange, collaboration and communication, and more comprehensive care.^78^

## Strengths and limitations

A strength of this study is that we have provided a broad insight into the current practice of palliative care in primary care and nursing homes, two settings with a high need for palliative care and, from a Dutch coordination of care model of palliative care, all HCPs should be able to provide this care. Several limitations of this study have to be taken into consideration. This was a cross-sectional study with a small sample, conducted in a defined region of the Netherlands in a convenience sample of HCPs working in primary care and five nursing homes. Primary care respondents were mainly members of a PaTz group (72%), where GPs and DNs meet to discuss patients with palliative care needs.^66^ These respondents are probably more aware of and experienced with palliative care. Moreover, only 4% of the primary care respondents were chaplains and 1% were physiotherapists. Although the perspective of these professions is underreported in this study, this reflects reality as they are infrequently involved in palliative care.^70^ A convenience sample was used, which can result in either an overestimation or an underestimation of bottlenecks, and as participation was voluntary, there could have been a selection bias. All HCPs were represented in the sample; therefore, the results can serve as a first broad understanding of current practice and guide the next steps to be taken in a large action research design study. There was a delay in publishing the study, but the situation is not expected to have changed. Due to the increasing pressure on health care in recent years, health care is rather underrated in this study. In this study, we did not use an existing, psychometrically tested survey instrument but developed one ourselves. The whole range of variables that may possibly influence palliative care may not have been fully explored, although face and content validity were tested by experts and professionals from all education levels.

## Practical implications

To improve the provision of palliative care, it is crucial to understand the current practice and possible needs of HCPs. Optimal palliative care can only be provided if patients with possible palliative care needs are identified. HCPs need to be aware of the palliative phase and changes that apply to it to deploy palliative care earlier. Furthermore, incorporating an increased use of marking instruments, such as the Surprise Question,^79^ and PROMS, such as the Utrecht Symptom Diary,^80^ should be facilitated, along with tools and education as to how to use them and how to engage in conversations with patients. A systematic approach to the provision of palliative care, including standardized notes and assessment tools for HCPs, should be implemented within organizations, in which multiprofessional consultations are included by default and where the electronic health record is adapted to this approach. Implementation of such an approach should include all HCPs of the multiprofessional team and be supported by management. For the implementation of such an approach to succeed, HCPs should be able to improve their capabilities and skills, which should begin within the curricula of the faculties and multiprofessional education.^81^ Once at work, organizations should create opportunities for continued learning from one another, interactively, in a team with the explicit goal of improving collaboration.^82^

## Conclusion

In primary care and nursing homes, barriers to the provision of palliative care exist. The provision of palliative care depends on the identification of patients with palliative care needs and is influenced by the knowledge and competencies of individual HCPs, possibilities for multiprofessional consultation, and optimal documentation in the electronic health record. An unambiguous and systematic approach is needed. Implementation of change should be patient-driven and tailored based on the variations in setting, profession, and educational level.

## Declarations

**Ethics approval and consent to participate:** The study was conducted in accordance with the principles of the Declaration of Helsinki and the General Data Protection Regulation. The Medical Research Ethics Committee in Utrecht, the Netherlands, confirmed that the Medical Research Involving Human Subjects Act did not apply [WAG/mb/19/010438]. HCPs were included based on their willingness to participate. Informed consent to participate was obtained from participants. The survey began with a study description, and completing the survey was considered informed consent. Data were collected anonynously and stored on a secured server accessible only by the research group.

**Consent for publication:** Not applicable.

**Availability of data and materials:** The data that support the findings of this review are available from the corresponding author upon request.
